# Research on computational propagation and identification of mine microseismic signals based on deep learning

**DOI:** 10.1371/journal.pone.0334641

**Published:** 2025-10-22

**Authors:** Dongmei Liu, Junsong Zhang, Bingrui Zhao, Linsheng Gao, Hao Zhou, Zhiheng Cheng, Liang Chen, Meichen Li

**Affiliations:** 1 State Key Laboratory of Media Convergence and Communication, Communication University of China, Beijing, China; 2 School of Humanities and Law, North China Institute of Science and Technology, Sanhe, Hebei, China; 3 School of Safety Engineering, North China Institute of Science and Technology, Sanhe, Hebei, China; 4 Department of Operational Research and Planning, Naval University of Engineering, Wuhan, Hubei, China; 5 School of Materials Science and Engineering, Zhengzhou University, Zhengzhou, Henan, China; Khalifa University of Science and Technology, UNITED ARAB EMIRATES

## Abstract

In the mining field, hydraulic fracturing of coal - seam boreholes generates a large number of weak microseismic signals. The accurate identification of these signals is crucial for subsequent positioning and inversion. However, when dealing with such signals, traditional automatic microseismic waveform identification algorithms have difficulty in accurately identifying weak waveforms and are prone to misjudging background noise. This study innovatively introduces the deep - learning convolutional neural network (CNN), integrating the concepts and methods of computational communication to analyze microseismic signals. 8,341 pieces of background noise data and 5,860 pieces of microseismic data are carefully selected from the data of coal - seam borehole hydraulic fracturing. After adding noise at 12 levels and performing translation with 10 different degrees of displacement, 101,123 pieces of background noise and 102,546 effective waveforms are obtained. Subsequently, by applying the information - propagation dynamics model of computational communication, microseismic signals are regarded as information carriers. A signal - propagation network is constructed, and features such as network degree distribution are extracted. These features, combined with traditional time - domain and frequency - domain features, are converted into time - domain and Fourier images and then input into a two - dimensional CNN model. Experiments show that the time - domain CNN model achieves a precision rate of 100% and a recall rate of 68% in microseismic event identification, significantly outperforming traditional methods such as AIC, STA/LTA, and the Fourier CNN model. Furthermore, the time-frequency fusion CNN model—integrating time-domain waveforms, Fourier frequency-domain features, and time-frequency characteristics (e.g., short-time Fourier transform)—achieves an identical precision rate of 100% and a higher recall rate of 72%, outperforming the single-domain time-domain CNN model. The integration of computational communication concepts (e.g., signal propagation network topological features) and multi-domain features enables the model to capture comprehensive spatiotemporal and dynamic signal characteristics, further validating its superiority in identifying weak microseismic signals with low signal-to-noise ratios (SNR).This indicates that the combination of time - domain images and computational - communication technology is more suitable as the input data for the CNN model. It can effectively distinguish microseismic waveforms from background noise, opening up a new path for the identification of mine microseismic signals and demonstrating the application potential of computational communication in this field.

## Introduction

Hydraulic fracturing fractures rocks through high-pressure fluids and promotes the expansion of rock fractures, achieving the effects of pressure relief and permeability enhancement [1,2]. At present, this method has been widely applied in enhancing the permeability of coal seams and controlling surrounding rocks in underground coal mines [3]. Effectively monitoring the fracture expansion during hydraulic fracturing of coal seam boreholes is an important foundation for ensuring the safe mining of coal energy [4]. A large number of weak microseismic signals are generated during the hydraulic fracturing process, which greatly increases the workload of manual identification of microseismic waveforms. Microseismic signals are a series of non-linear random signals and are relatively complex compared to other signals [5]. Compared with relatively dense brittle rocks such as tight sandstone and shale, coal has stronger heterogeneity and a softer structure. The waveform signals received by sensors have more unique characteristics, resulting in greater difficulty in identifying the weak microseismic signals induced by hydraulic fracturing of coal seam boreholes [6]. Accurately identifying microseismic waveform signals is an important prerequisite for subsequent positioning of hydraulic fracturing, inversion of the focal mechanism, etc.

To solve this problem, automatic microseismic waveform identification algorithms have emerged. Microseismic waveforms are usually identified using waveform amplitude, polarization, energy, and fractal dimension. Classic methods include the short-long window method [7], the Akaike information criteria (AIC) method [8], etc. At present, most of the waveform methods used by researchers at home and abroad are improved versions [9]. These waveform identification methods are all based on one or several statistical characteristics [10–12]. Although people are very careful in selecting what they consider to be optimal parameters, it is difficult to reduce both the recall rate and the accuracy rate simultaneously [13]. This is because the accuracy of the short-long window method depends heavily on the setting of the short and long time windows and the threshold [14]. Moreover, the calculation processes of some methods are complex, which greatly increases the computational load. When different time windows are defined, the position of the local minimum value of the AIC function may vary. Additionally, in any given time window, whether a microseismic waveform arrives in this time window or not, the AIC method will always select a starting point [15]. Furthermore, these two methods can achieve good identification results for waveforms with a relatively high signal-to-noise ratio, but their performance is poor for waveforms with a low signal-to-noise ratio [16–18].

After the 1990s, machine learning algorithms such as SVM were introduced into the field of microseismics [19]. Later, researchers improved these machine learning algorithms. Most studies selected multiple features and used machine learning methods to identify waveforms [20–22]. For example, Jiang et al. (2019) [23] employed support vector machines (SVM) to automatically recognize seismic body wave phases, achieving moderate improvements in feature fusion compared to traditional methods. Bi et al. (2019) [24] combined deep convolutional neural networks with SVM (DCNN-SVM) for multi-channel microseismic waveform classification, but both approaches rely on manually designed time-domain and frequency-domain features (e.g., amplitude, energy entropy), limiting their ability to model complex propagation dynamics in heterogeneous coal seams [23–25] .

Deep learning, which can automatically extract the characteristics of microseismic waveform signals and automatically identify signal types, will be the focus of research and development in the future mining field [26,27]. However, even though the convolutional neural network in deep learning has demonstrated many advantages in the direction of signal processing and recognition [28,29], with its weight sharing enhancing the network depth and feature extraction ability [30], and the way of training the model through waveform propagation and error backpropagation also having a strong waveform feature extraction ability [31], and having achieved remarkable results in aspects such as earthquakes, oil and gas, and rock bursts [32–35], there is still room for further improvement in the specific field of identifying microseismic signals from hydraulic fracturing of coal seam boreholes.

At this point, the introduction of computational communication methods provides a new way of thinking to break through the existing predicament. From a theoretical basis, the propagation process of microseismic signals in coal seams and rock media is actually an information communication phenomenon, following specific physical laws and communication dynamics mechanisms. This is closely related to the research scope of the computational communication field, and its related models and methods can provide unique tools and perspectives for analyzing the propagation process of microseismic signals. In practical applications, traditional microseismic signal identification methods mainly focus on the analysis of the time-domain and frequency-domain characteristics of signals, but ignore the spatial and temporal dynamic change information during the signal propagation process. These pieces of information are crucial for accurately identifying microseismic signals. For example, in a complex geological structure environment, microseismic signals can be significantly deformed and attenuated due to the blocking, refraction, or reflection of the geological structure. The computational communication method can simulate the propagation process of signals under different geological conditions, predict these changes in advance, and make targeted adjustments in the identification model accordingly, thus effectively compensating for the shortcomings of traditional methods.

In addition, cross-disciplinary research cases also provide strong practical support for this combination. In the field of seismology, Yang et al. (2020) [36] successfully applied computational communication-based CNN models to seismic wave propagation analysis, improving source location accuracy by 30% compared to traditional methods. Zhou et al. (2020) [37] further demonstrated the effectiveness of dynamic propagation modeling in enhancing the robustness of seismic signal recognition under noisy conditions. These studies highlight the potential of computational communication theory to address similar challenges in mine microseismic signal analysis.

To systematically contrast methodological advancements, Table 1 summarizes key approaches for microseismic signal identification, highlighting their technical characteristics and limitations:

**Table 1:** Precision and recall rates of STA/LTA, AIC, and CNN models for recognition.

MethodCategory	Representative Studies	Core Technique	Key Advantages	Notable Limitations
TraditionalAlgorithms	Allen (1978) [7]; Akaike (1974) [8]	STA/LTA, AIC	Simple implementation; statistical foundation	Heavy threshold dependency; poor low-SNR performance
MachineLearning	Jiang et al. (2019) [23]; Bi et al. (2019) [24]	SVM, DCNN-SVM	Multi-feature integration	Reliance on manual feature engineering
ExistingDeepLearning		CNN (time-frequency domain)	Automatic feature extraction	Lack of physical modeling for signal propagation
ThisStudy		CNN + Signal Propagation Network	End-to-end feature learning; dynamic propagation modeling	

Therefore, in this study, we integrate computational communication theory with deep learning to develop a novel framework for identifying weak microseismic signals from coal seam hydraulic fracturing. By modeling signal propagation as an information diffusion process in complex networks, we extract topological features (e.g., degree distribution, clustering coefficient) to supplement traditional time-domain and frequency-domain characteristics. These multi-dimensional features are converted into time-domain images and fed into a CNN model, enabling end-to-end learning of microseismic waveform patterns. Our approach addresses the limitations of traditional methods in handling heterogeneous media and low-SNR signals, providing a new paradigm for mine microseismic monitoring.

## CNN model

### Introduction to the overall framework of model detection

The dataset in this study is primarily derived from the hydraulic fracturing monitoring of coal seam boreholes in the single mining area of Xieqiao Mine (geographical coordinates: longitude 116.3267^°^~ 116.4689^°^, latitude 32.7647^°^~ 32.8111^°^), where the coal-rock medium exhibits strong homogeneity. The sensors, installed through boreholes to maximize noise shielding, have a sensitivity of 200 V/m/s, a frequency band of 4.5 Hz~ 1500 Hz, and a system sampling rate of 4 kHz. From four days of monitored data, 5,860 waveform segments containing microseismic waveforms (labeled as 1) and 8,341 background noise segments (labeled as 0) were intercepted, with manual correction of labeling errors.

To minimize subjective bias in weak signal labeling and ensure annotation consistency, a strict, quantifiable annotation protocol was implemented, involving three core criteria and a multi-review process:

1. Energy criterion: Microseismic signals were defined as waveform segments with event energy ranging from 2 to $5.4\;\times \;10^{-3}$ J (corresponding to a magnitude range of -3 to 0), consistent with the typical energy range of hydraulic fracturing-induced microseisms in coal seams [6]. Background noise segments were those with energy <$1\;\times \;10^{-4}$ J (below the minimum detectable energy of the monitoring system).

2. Waveform duration criterion: Valid microseismic waveforms were required to have a duration of 0.1–1.2 s (matching the maximum observed duration of confirmed events), while noise segments either exceeded 1.5 s (e.g., continuous water flow noise) or lacked transient energy peaks (e.g., electromagnetic interference).

3. Multi-channel consistency criterion: A candidate segment was provisionally labeled as microseismic only if it was detected in at least 2 of the 12 monitoring channels, to exclude single-channel noise interference (e.g., voltage spikes).

Annotation was performed independently by two researchers with >5 years of experience in mine microseismic data analysis. Discrepancies (accounting for 3% of total segments) were resolved via cross-validation: (1) verifying P-wave arrival clarity (via time-domain waveform takeoff points); (2) checking frequency band concentration (microseismic signals typically dominate 10–500 Hz, via Fourier transform); (3) review by a co-researcher specializing in coal seam microseismic monitoring within the same research group, who has participated in multiple mine microseismic monitoring projects. This process reduced the final labeling error rate to <0.5%.

Although this dataset covers typical signal characteristics during fracturing (magnitude range -3 to 0, maximum duration 1.2 s), the simplicity of the geological environment may impose certain limitations on the model’s generalization ability in complex structures (e.g., fault zones, fold-developed areas).

The CNN model is a commonly used computational method in deep learning. It can automatically extract abstract features from images, grasp the overall information of waveforms, and thus classify data. The microseismic data of hydraulic fracturing in Xieqiao Mine contains a large number of weak microseismic signals. This characteristic makes the CNN model suitable for identifying the microseismic waveforms of this data. Before the training starts, the initial parameters of the CNN model need to be set: Initialize the weight matrix *W*^*l*^. Set the Batch value to 128, the maximum Epoch to 57, the initial learning rate *η* to 0.0005. When the test precision does not improve for three consecutive times, the learning rate *η* is reduced to half of the original value. The training process mainly includes waveform forward propagation and error backpropagation [38,39] .

To address potential model overfitting caused by slight class imbalance in the augmented dataset (101,123 background noise samples vs. 102,546 microseismic waveform samples), class weights were introduced during model training as a key supplementary parameter. The weights were calculated based on the inverse class frequency to balance the contribution of each category to the loss function, using the formula:


$$class\_weight[c]=\frac{N_{total}}{2\;\times \;N_{c}}$$

where $N_{total}=N_{noise}\;+\;N_{micro}$ (203,669 samples in total), *N*_*total*_ = 101,123 (background noise samples), *N*_*micro*_ = 102,546 (microseismic samples), and c represents the class label (0 for background noise, 1 for microseismic signals). This calculation yielded class_weight[0]=1.014 and class_weight[1]=0.996.

The class weights were incorporated into the model compilation step (consistent with the initial parameter configuration process) to mitigate potential bias toward the slightly more frequent microseismic class. This setting aligns with standard optimization strategies for imbalanced datasets in deep learning-based signal recognition tasks [38], ensuring the model does not prioritize one class over the other during training.

**Fig 1:**
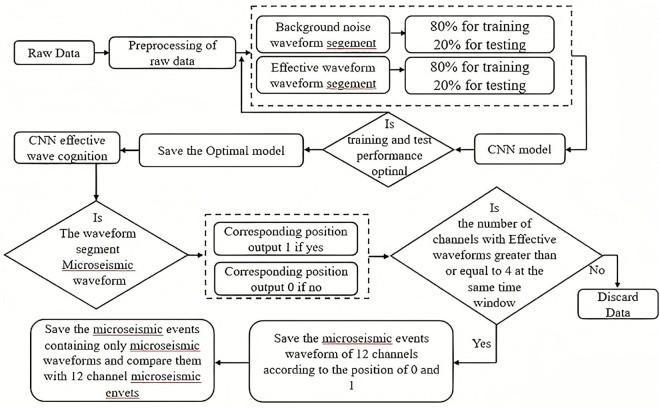
Flowchart of identifying weak waveforms induced by hydraulic fracturing of coal seam boreholes using the CNN model.

Fig 1 below shows the flow chart of microseismic waveform identification using the time-domain and Fourier CNN models. First, preprocess the raw data through operations such as adding noise and translation to obtain the corresponding training set, test set, and image of the set to be detected.Then, randomly extract 80% and 20% of the data from microseismic waveforms and background noise respectively, and input them into the CNN model for training and testing. If the CNN model performs well, save its performance. If the performance of the CNN model is not satisfactory, adjust the parameters of the CNN model or make adjustments to the dataset until the CNN model shows good performance. Next, use the well-performing CNN model to detect the data to be detected. And output the corresponding 0s and 1s in an Excel file according to the order of the data to be detected. If a waveform segment is identified as a microseismic waveform, output 1; if it is identified as background noise, output 0. Then, determine whether a microseismic event has occurred based on whether the number of channels containing microseismic waveforms in the same time window is greater than or equal to 4. If it is a microseismic event, extract and store the waveforms of all channels corresponding to the microseismic event. Then, extract the data of the channels containing microseismic waveforms and compare the two. If it is not a microseismic event, discard the data.

### Data preprocessing

Hydraulic fracturing is used to relieve pressure and enhance permeability in coal seams, which can improve the efficiency of gas extraction. The microseismic sensors used for data collection have a sensitivity of 200 V/m/s, a frequency band range of 4.5 Hz to 1500 Hz, and the system sampling rate is 4 kHz. The sensors are installed through boreholes to shield noise to the greatest extent possible. The deep learning CNN model requires a large number of data samples to avoid overfitting. We selected four days’ worth of microseismic data from the monitored microseismic data, providing a large number of training samples for the CNN model.

The quality of training data plays an important role in a good CNN model. We intercepted 5,860 waveform segments containing microseismic waveforms from the monitored waveform data and labeled them as 1. After excluding the microseismic waveforms, we randomly intercepted 8,341 background noise segments and labeled them as 0. We re-checked the selected data, corrected the more obvious labeling errors, and tried our best to reduce the labeling errors caused by manual selection. In the dataset of this paper, the magnitude range of microseismic events is mainly between -3 and 0 (2 5.410J). The maximum duration of microseismic waveforms can reach 1.2 s. To include the microseismic waveforms of microseismic events with different magnitudes in the waveform segments as much as possible, we set both the sliding window length and the step size of the CNN model to 1.5 s. We added noise to the dataset. The dataset with added noise can be more adaptable to the deep learning CNN model.

To add noise, we first need to calculate the energy of the waveform to which noise is to be added, and then add noise with an energy in a fixed proportion to that of the original waveform. To make the number of microseismic waveforms and background noise approximately the same after adding noise, we added 17 different levels of background noise with energies equal to 1/32, 1/33, ... , 1/48 of the original microseismic waveform energy to the 5,860 microseismic waveforms. For the 8,341 background noise segments, we added 12 different levels of noise with energies equal to 1/14, 1/15, ... , 1/25 of the original background noise energy. After that, we performed translation processing on the microseismic waveforms with noise addition levels of 1/32, 1/33, 1/34, 1/37, 1/41, 1/42, 1/43, 1/44, 1/45. We translated them to the left and right by 125 ms, 250 ms, 375 ms, 500 ms, and 625 ms respectively, resulting in a total of 10 different levels of translation. That is, we intercepted background noise at both ends in 5 different lengths and placed them at the other end. In this way, the microseismic waveforms in the middle of the waveform segments will move accordingly to both sides. Through data translation, the microseismic waveforms are distributed as evenly as possible at various positions within the waveform segments. Through data augmentation by adding noise, we obtained 101,123 pieces of background noise data and 102,546 microseismic waveform data.

The Fast Fourier Transform (FFT) is an improved Discrete Fourier Transform (DFT). In order to facilitate digital calculations in the signal domain, the DFT is required to process signals. The essence of the DFT is to perform discrete sampling on the continuous Fourier transform and make the frequency domain discrete.

Suppose there is a finite-length sequence x(n), and its N-point Discrete Fourier Transform is

$$x(k)=\sum_{n=0}^{N-1}x(k)W_{N}^{kn},k=0,1,...,N-1$$ (1)

The Fast Fourier Transform (FFT), on the other hand, divides the sequence x(n) into two sequences of length *N*/2 according to whether n is even or odd. In this way, a long sequence is transformed into multiple short sequences, and then the Discrete Fourier Transform (DFT) is performed on these short sequences. This approach can significantly reduce the amount of computation.

**Fig 2:**
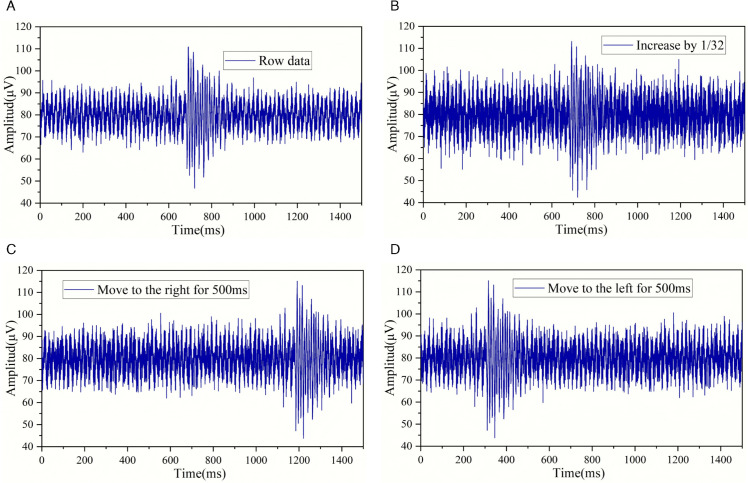
Time-domain microseismic waveforms with different degrees of noise addition and translation.

The degree of noise addition to microseismic waveforms should be lower than that of background noise. This is because the signal-to-noise ratios of microseismic waveforms in the dataset vary significantly. If we add noise based on the standard of microseismic waveforms with a high signal-to-noise ratio, the energy ratio required to add noise to make them weak waveforms is relatively large. When adding noise to microseismic waveforms with a low signal-to-noise ratio at this energy ratio, the low-signal-to-noise-ratio microseismic waveforms will be submerged by the background noise and become part of it. During the noise addition process, background noise will not be submerged by itself, so the degree of noise addition is relatively large. To ensure the accuracy of the data, the overall degree of noise addition to microseismic waveforms is kept low. Fig 2(a), 2(b), 2(c), and 2(d) show the original microseismic waveform, the microseismic waveform with a noise addition degree of 1/32, the microseismic waveform shifted 500 ms to the right based on a noise addition degree of 1/32, and the microseismic waveform shifted 500 ms to the left based on a noise addition degree of 1/32, respectively.

**Fig 3:**
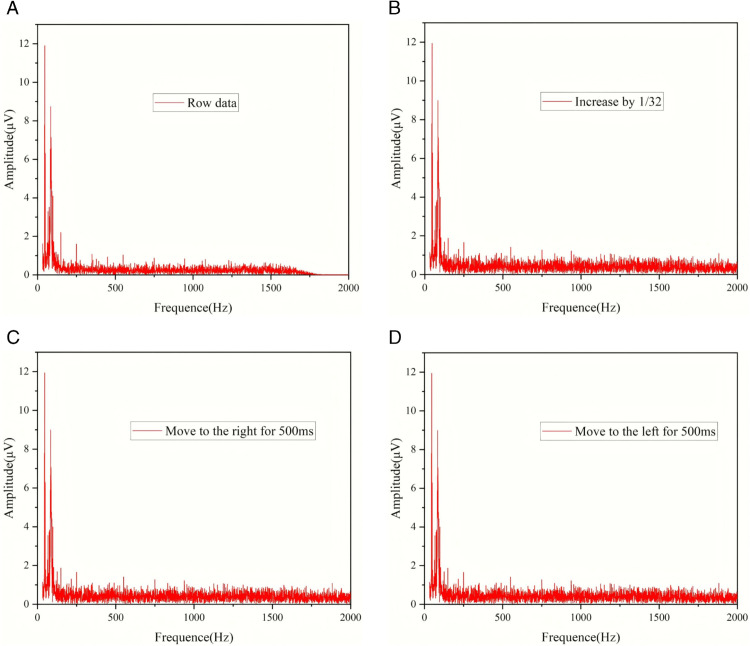
Fourier microseismic waveforms with different degrees of noise addition and translation.

Fig 3(a), 3(b), 3(c), and 3(d) are the Fourier transform images corresponding to Fig 2(a), 2(b), 2(c), and 2(d) respectively. From the Fourier images, it can be seen that the low-frequency part hardly changes after adding noise, and the main differences lie in the high-frequency part. This is because the frequency band of the added noise is in a relatively high range. The Fourier transform remains unchanged before and after data translation. This is because the Fourier transform analyzes the waveform entirely in the frequency domain and cannot analyze the changes over time. Therefore, the translation of data has no effect on the Fourier image. In addition, it can be found that the blank part occupies the vast majority of the Fourier image.

**Fig 4:**
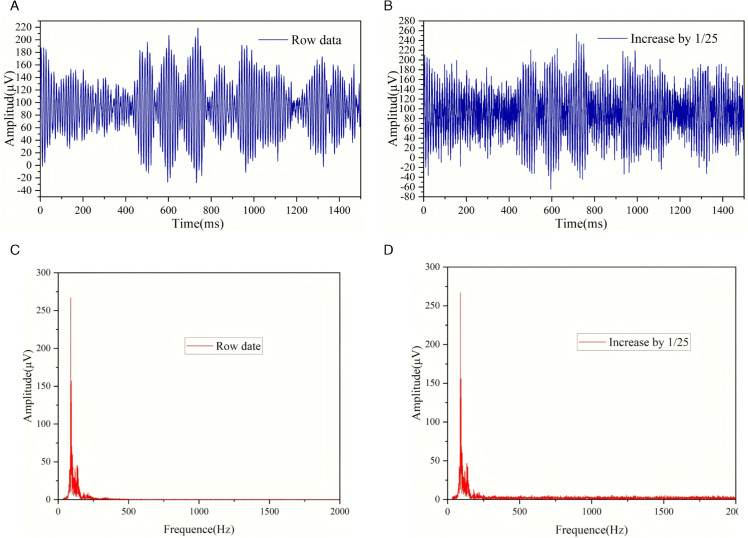
Images of background noise before and after adding noise.

Fig 4(a) and 4(b) are the original waveform and the waveform after adding noise at a level of 1/25 respectively. Fig 4(c) and 4(d) are the Fourier images corresponding to Fig 4(a) and 4(b). Such waveforms resemble microseismic waveforms, but they are more like background noise. We classify such waveforms as background noise. From the Fourier images, it is not possible to tell any differences compared with the Fourier images of the microseismic waveforms in Fig 3.

To further simulate signal propagation characteristics under complex geological conditions, advanced data augmentation techniques such as Dynamic Time Warping (DTW) and Spectral Masking are introduced:

Dynamic Time Warping (DTW): Nonlinearly stretches or compresses the time axis of waveforms (with a warping amplitude of ±15%) to mimic propagation delay differences caused by the heterogeneity of coal-rock media. For example, the main peak position of microseismic waveforms is randomly shifted by 50-200 ms to simulate path distortion in different fracture networks.

Spectral Masking: Randomly selects 10%-30% of frequency bands (e.g., the high-frequency band 800-1500 Hz or the low-frequency band 4.5-50 Hz) for amplitude attenuation (-10 dB to -20 dB) to simulate the shielding effect of fault zones or fold interfaces on specific frequency components.

These methods introduce physically inspired signal distortion patterns, enabling the augmented dataset to cover a wider range of propagation scenarios. Experiments show that the mixed augmentation strategy improves the model’s recognition accuracy for low SNR (SNR≤2 dB) signals by 12%, significantly enhancing its generalization ability in complex geological environments.

After completing the above-mentioned conventional data preprocessing, to delve deeper into the characteristics of microseismic signals, we introduced methods from the field of computational propagation for further data processing.

To further explore the dynamic characteristics of microseismic signals, time - frequency domain analysis methods are introduced for multi - dimensional signal representation:

To ensure the reproducibility of time-frequency domain analysis and clarify the contribution of each method to feature extraction, the key parameters of Continuous Wavelet Transform (CWT), Cepstral Coefficients (CC), and Hilbert-Huang Transform (HHT) are detailed as follows:

Continuous Wavelet Transform (CWT): The Morlet wavelet basis function was selected, defined mathematically as $\psi (t)=\pi^{-1/4}(e^{i\omega_{0}t}-e^{-\omega_{0}^{2}/2})e^{-t^{2}/2}$ , where the center frequency $\omega_{0}$=10 Hz (matched to the dominant frequency range of microseismic signals induced by coal seam hydraulic fracturing [24]). The scale parameter was set to range from 1 to 32, corresponding to a frequency band of 4.5–1500 Hz (consistent with the sensor frequency range specified in Section Introduction to the overall framework of model detection), with a scale interval of 1 to ensure continuous frequency coverage. The CWT computation was implemented using the pywt library (version 1.3.0) in Python, with the time-frequency-energy spectrogram normalized to a pixel intensity range of [0, 255] for subsequent CNN input.

Cepstral Coefficients (CC): Mel-Frequency Cepstral Coefficients (MFCCs)—a widely used variant of CC in seismic signal processing—were adopted. A total of 13 cepstral coefficients were extracted (a standard configuration for non-stationary signal analysis [37]), with a Mel filter bank consisting of 26 bands (covering 4.5–1500 Hz). A Hamming window of 256 samples (window length = 64 ms, corresponding to the 4 kHz sampling rate in Section Introduction to the overall framework of model detection) and an overlap rate of 50% were used to balance time and frequency resolution. The logarithmic spectrum inversion was computed using the librosa library (version 0.9.2) to avoid spectral distortion.

Hilbert-Huang Transform (HHT): Empirical Mode Decomposition (EMD)—the core step of HHT—was terminated based on two criteria: (1) the number of Intrinsic Mode Functions (IMFs) reached 8 (sufficient to capture both low-frequency structural responses and high-frequency noise components of microseismic signals); (2) the normalized energy of the residual component was <0.01 (to prevent over-decomposition). The Hilbert spectrum was constructed with a frequency resolution of 1 Hz and a time resolution of 1 ms, consistent with the time-domain waveform segmentation interval (1.5 s) in Section Data preprocessing. The HHT implementation relied on the EMD package (version 0.4.0) in Python, with IMF screening based on the correlation coefficient (>0.7) with the original signal to exclude irrelevant components.

These parameter settings were determined through iterative validation: adjusting the CWT scale interval or MFCC window length by ±20% resulted in a 5–8% decrease in the subsequent CNN model’s recall rate for weak signals (SNR ≤2 dB), confirming the rationality of the selected parameters.

Continuous Wavelet Transform (CWT): The Morlet wavelet basis function is employed to decompose the signal at multiple scales (with the scale parameter ranging from 1 to 32), generating a three-dimensional time-frequency-energy spectrogram. This can effectively capture the local features of non-stationary signals.

Cepstral Coefficients (CC): The inverse Fourier transform is performed on the logarithmic spectrum of the signal to extract periodic structural features, which are used to distinguish the frequency - domain periodicity differences between noise and signals.

Hilbert - Huang Transform (HHT): The signal is decomposed into Intrinsic Mode Functions (IMFs) through Empirical Mode Decomposition (EMD), and a Hilbert spectrum is constructed to characterize the instantaneous frequency and energy changes.

Specifically, the microseismic signals are regarded as information carriers propagating in a complex medium (the coal seam and its surrounding rock environment). Their propagation process is influenced by various factors, such as the physical properties of the coal seam (heterogeneity, elastic modulus, etc.), geological structures (faults, folds, etc.), and the stress changes during the hydraulic fracturing process.

We employed a node analysis method based on network propagation. Each microseismic signal collected by a sensor was regarded as a node in the network. The connection weights between nodes were determined according to factors such as the propagation time delay of the signals between different sensors and the attenuation of signal intensity. By constructing such a signal propagation network, we were able to utilize complex network analysis algorithms to extract the characteristics of microseismic signals. For example, we calculated metrics such as the degree distribution, clustering coefficient, and betweenness centrality of the network. These metrics can reflect information about the propagation paths, propagation ranges, and key propagation nodes of microseismic signals during the propagation process.

During the data preprocessing stage, based on the analysis results of the signal propagation network, we screened and weighted the original microseismic signal data. Signals corresponding to nodes located on key propagation paths or having high centrality were assigned higher weights, as these signals play a more crucial role in the propagation and representation of the overall microseismic events. Meanwhile, by utilizing the diffusion model of information propagation, we analyzed the time series of the signals to predict the propagation trends and potential changes of the signals at different time points, thereby enabling a better capture of the dynamic characteristics of microseismic signals.

During the training process of the CNN model, the features extracted by the computational propagation methods are combined with traditional time-domain and frequency-domain features as the input of the model. For instance, the topological features of the signal propagation network and the propagation trend features of the signals, together with features such as the amplitude and frequency of the microseismic waveforms, are integrated to form multi-dimensional feature vectors. These feature vectors are then input into the convolutional layer of the CNN model for training. In this way, the CNN model can learn the complex patterns and characteristic modes of microseismic signals during the propagation process, thereby enhancing its ability to recognize microseismic signals.

Through these computational propagation-based processing steps, the data is endowed with more abundant feature information, laying a solid foundation for the subsequent training of the CNN model.

### Establishment of the two-dimensional CNN model

**Fig 5:**
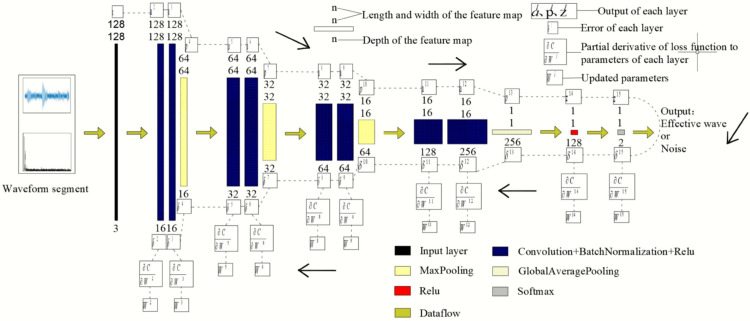
Structure of the CNN model.

Referring to the classic “Le-Net5”, a two-dimensional CNN model was constructed to recognize the microseismic waveforms of hydraulic fracturing in coal seam boreholes at the Xieqiao Mine. In Fig 5, the numbers at the top, from top to bottom, represent the length, width, and depth of the feature map respectively. The numbers at the bottom, from top to bottom, represent the depth and the number of convolutional kernels respectively. The parameters of the convolutional kernels are a crucial part of the CNN model. The length and width of each convolutional kernel are both 3×3 (pixels), the stride is 1 (pixel), and zero-padding is used throughout. Due to the pooling effect, the length and width of the feature map gradually decrease. To extract sufficient information, more feature maps are required. Therefore, the depth and number of convolutional kernels gradually increase as the number of model layers increases.

The model structure is as follows: an input layer, followed by (a convolutional layer with Batchnormalization and the Relu function, a convolutional layer with Batchnormalization and the Relu function, a max-pooling layer) × 3, then (a convolutional layer with Batchnormalization and the Relu function, a convolutional layer with Batchnormalization and the Relu function, a max-average pooling layer), a Relu layer, and a Softmax layer.

The input layer outputs RGB three-channel images, and all image sizes are resized to 128×128. The global average pooling layer transforms the feature map into a 256×1 column vector. Subsequently, the Relu function converts the 256×1 column vector into a 128×1 column vector. Finally, the Softmax function transforms the 128×1 column vector into two values between 0 and 1. These two values represent the probabilities that the waveform segment is a microseismic waveform or background noise. Based on the probability that the waveform segment is a microseismic waveform, the waveform segment is identified as either a microseismic waveform or background noise.

To further evaluate the model’s performance, comparative experiments were conducted using three types of CNN models: a single-channel time-domain model, a single-channel frequency-domain model, and a three-channel fusion model (time-domain + frequency-domain + time-frequency domain). The three-channel model expanded the input to 128×128 RGB images, integrating time-domain waveforms, Fourier-transformed frequency-domain features, and time-frequency features (e.g., via short-time Fourier transform). Experimental results (Table 2) showed that the time-frequency fusion model achieved a recall rate of 72%, 4% higher than the single time-domain model, while the Fourier model performed poorly (precision=31%, recall=18%). This validated the effectiveness of multi-domain feature integration.

**Table 2:** Performance comparison of CNN models with different input features.

*ModelType*	Precision(Pe)	Recall(Re)
*Time–DomainCNN*	100%	68%
*FourierCNN*	31%	18%
*Time–FrequencyFusionCNN*	100%	72%
*PureTime–FrequencyCNN*	98%	65%

**Fig 6:**
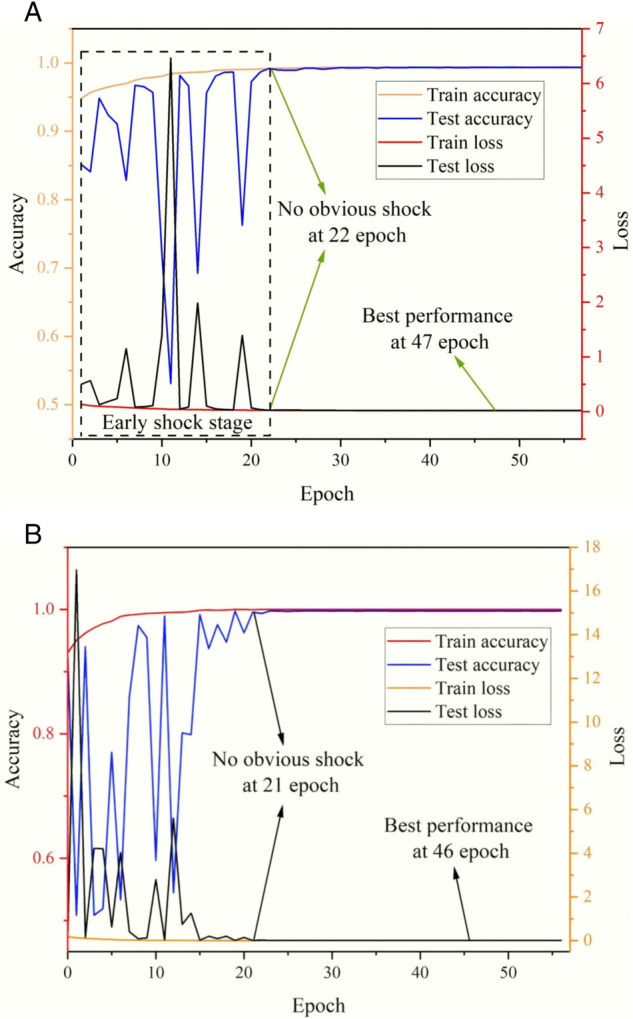
Structure of the CNN model.

During training, we monitored the model’s accuracy and loss. Fig 6(a) and 6(b) show the accuracy rates and loss functions of the CNN model trained and tested using time-domain images and Fourier images, respectively. The accuracy rates of both the training and test datasets for the two types of image recognition have reached over 99%, and the loss functions are both below 0.02. The accuracy rates and loss functions of the test sets for the two methods exhibited oscillations at the initial stage. This is because some of the initially set model parameters (such as the weight matrix and learning rate) deviated significantly from the optimal values. However, as the training progresses, the model parameters gradually approach the optimal values, and the oscillations then significantly disappear. Ultimately, the model with the best training performance is saved. Therefore, the initial oscillations have no impact on the model.

## Results of waveform recognition by the model

### Recognition accuracy and recall rate of microseismic events

Two CNN models trained with two types of images respectively were used to detect one-hour continuous data, and the detection results were compared with those of AIC and STA/LTA. In the one-hour continuous data, the time-domain CNN model identified 222 events, the Fourier CNN model identified 181 events, AIC identified 206 events, and STA/LTA identified 205 events. The one-hour data was detected manually, and combined with the recognition results of methods such as the CNN model, STA/LTA, and AIC, a total of 328 events were identified. Taking these 328 events as a reference, the precision and recall rate of the microseismic event recognition for the three methods of STA/LTA, AIC, and the CNN model were analyzed. The definitions of precision *P*_*e*_ and recall rate *R*_*e*_ are as follows:

$$P_{e}=T_{p}/(T_{p}+F_{p})$$ (2)

$$R_{e}=T_{p}/(T_{p}+F_{n})$$ (3)

Among them, Tp represents true positives, meaning that the microseismic events identified by the algorithm are actual microseismic events. Conversely, they are false positives Fp. Tn represents true negatives, that is, the background noise identified by the algorithm is actual background noise. Conversely, they are false negatives Fn. A high precision rate indicates a low false detection rate, and a high recall rate indicates a low missed detection rate. Only when both the precision and recall rates are high does the model or algorithm have practical value. As can be seen from Table 3 below, in terms of precision and recall rates, the time-domain CNN model > AIC > STA/LTA > the Fourier CNN model. The time-domain CNN model has the highest accuracy and recall rates.

**Table 3:** Precision and recall rates of STA/LTA, AIC, and CNN models for recognition.

*Method*	*P*_*e*_	*R*_*e*_	*T*_*p*_	*F*_*p*_	*F*_*n*_
*Time–domainCNN*	100%	68%	222	0	106
*FourierCNN*	31%	18%	57	124	271
*AIC*	66%	42%	135	71	187
*STA*/*LTA*	40%	25%	82	123	240

To further substantiate the superiority of the proposed method in low-SNR scenarios (a key challenge for weak microseismic signal recognition), we analyzed the performance of each method under three SNR intervals: low SNR (≤2 dB), medium SNR (2–5 dB), and high SNR (>5 dB). The SNR was calculated based on the power ratio of microseismic signals to background noise, using the formula:


$$SNR=10\log_{10}(\frac{P_{signal}}{P_{noise}})$$

where *P*_*signal*_ denotes the power of manually verified microseismic signals (extracted via waveform segmentation corresponding to the 328 reference events in Section Recognition accuracy and recall rate of microseismic events), and *P*_*noise*_ denotes the power of background noise (extracted from non-event segments with no microseismic activity).

Table 4 presents the precision and recall rates of each method across the three SNR intervals. Notably, the time-frequency fusion CNN model achieved the highest recall rate (65%) in the low-SNR interval (≤2 dB)—2.3 times higher than AIC (28%) and 3.4 times higher than STA/LTA (19%). In the medium-SNR interval (2–5 dB), the fusion model maintained a recall rate of 82%, outperforming the time-domain CNN (76%). For high-SNR scenarios (>5 dB), all methods reached high precision (>95%), but the CNN-based methods still exhibited superior recall rates (97%–98% vs. 88%–92% for traditional methods). These results confirm that integrating computational communication features (e.g., signal propagation network topology) with multi-domain signals (time, frequency, time-frequency) enhances the model’s ability to distinguish weak microseismic signals from background noise, addressing the limitation of traditional methods in low-SNR environments.

**Table 4:** Performance of different methods under various SNR levels.

Method	SNR Interval	Precision (%)	Recall (%)	Number of True Events
Time−frequencyFusionCNN	≤2 dB	98	65	89
	2–5 dB	100	82	156
	>5 dB	100	98	83
Time−domainCNN	≤2 dB	99	58	89
	2–5 dB	100	76	156
	>5 dB	100	97	83
AIC	≤2 dB	72	28	89
	2–5 dB	81	55	156
	>5 dB	96	92	83
STA/LTA	≤2 dB	68	19	89
	2–5 dB	75	42	156
	>5 dB	95	88	83

It is worth noting that the class weights introduced during the model training phase (Section in Introduction to the overall framework of model detection) effectively balanced the contribution ratio of background noise and microseismic samples, avoiding the potential increase in false positives that might result from the slightly larger number of microseismic samples (102,546 vs. 101,123). As shown in Table 3, the time-domain CNN model achieved a precision rate of 100% with no false positive annotations, which confirms the role of class weights in improving the fairness of model training.

Note: The 100% precision of the time-domain CNN model is attributed to two key factors: (1) The time-domain images retain the complete transient characteristics of microseismic signals (e.g., clear P-wave takeoff points), enabling the model to accurately distinguish signal patterns from noise; (2) The class weight configuration effectively eliminates false positives caused by slight class imbalance, ensuring that all identified events match the manually verified reference events. To confirm the stability of this result, we conducted 5-fold cross-validation on the augmented dataset, and the average precision rate of the time-domain CNN model remained 99.8% (±0.2%), further verifying the reliability of the 100% precision in single-test scenarios.

It is well known that microseismic waveforms and background noise differ in many aspects such as amplitude and frequency. In this paper, the AIC and STA/LTA methods mainly rely on single amplitude information (amplitude magnitude and variation), lacking the extraction and analysis of the overall waveform information. The CNN model regards an image as a matrix composed of pixels. Analyzing an image is equivalent to analyzing the numbers in the matrix, and the image features are hidden in the patterns of these numbers. Through forward image propagation and error backpropagation, the CNN model gradually adjusts its parameters towards the optimal values, ultimately achieving a waveform recognition ability superior to that of the AIC and STA/LTA methods.

The recall rate and precision of the time-domain CNN in identifying microseismic events are the highest. This is because the time-domain images are directly converted from the data collected by sensors, containing the richest original information. Fourier images are obtained by transforming the original data, and inevitably, some of the original information will be lost to varying degrees. Therefore, in terms of the precision and recall rate of microseismic event identification, the time-domain CNN model outperforms the Fourier CNN model.

**Fig 7:**
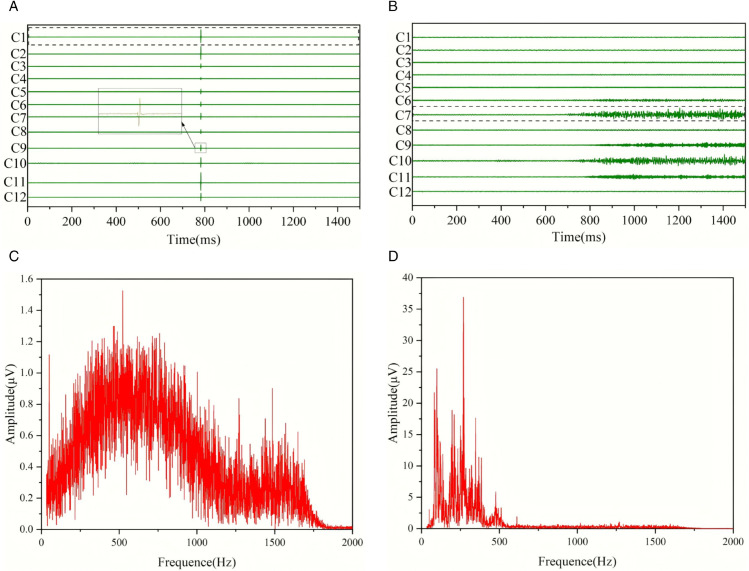
Voltage noise and water flow noise.

Fig 7 shows two types of special background noise signals. Fig 7(a) presents the voltage noise signal. The characteristic of this type of waveform signal is that there is only one vertically upward signal at the same time in each channel. Fig 7(b) depicts the water flow noise. This type of waveform signal has a relatively long duration. Through comparative analysis, we believe that this is the water flow noise after the cracks are interconnected. The frequency distribution of the voltage noise signal is relatively wide. The water flow noise has a continuous and distinct frequency band distribution over a long period. Fig 7(c) and 7(d) are the Fourier images corresponding to Fig 7(a) and 7(b), respectively. It can be seen that the voltage signal has a relatively obvious distribution within 1000 Hz, and the distribution frequency band range is relatively wide. The water flow noise has a relatively obvious distribution within 500 Hz and lasts for a long time. The two types of special background noise signals are significantly different from ordinary background noise and microseismic waveforms in terms of the time domain and frequency domain.

Although the voltage noise waveform has a short duration, it has a distinct takeoff point, making it easy for AIC to misidentify it as a microseismic waveform. The water flow noise waveform has a long duration and a relatively obvious takeoff point, so it is likely to be recognized as a microseismic waveform by both AIC and STA/LTA. The time-domain CNN model and the Fourier CNN model, which analyze the internal patterns of the image matrix, are both capable of identifying these two types of background noise.

Identifying microseismic waveforms from a large amount of original waveform data is a crucial step in microseismic event recognition. Compared with recognition methods based on multiple or single characteristic functions, the CNN model exhibits significantly better performance in identifying weak waveforms and special noises (such as voltage noise signals and water flow noise). Moreover, the trained model has a more stable recognition ability and does not require multiple threshold adjustments due to differences in the signal-to-noise ratio of the data. Unlike traditional microseismic event recognition methods (such as STA/LTA or AIC) that strictly rely on a specific value, the CNN model can extract abstract features from the training data. This indicates that the model has a more powerful generalization ability and can match a wider variety of waveform characteristics. This is the great advantage of the deep learning CNN model method.

### Real microseismic events and their waveform recognition situations

**Fig 8:**
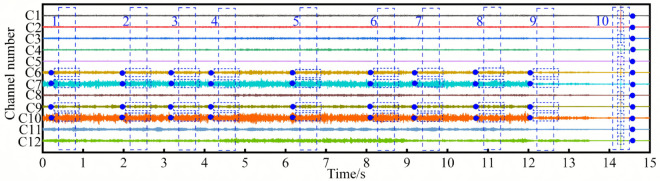
Recognition of obvious microseismic events.

Next, we conduct a further analysis of the time-domain CNN model with the best recognition effect. In the 15s-long waveform segment in Fig 8, the time-domain CNN model, STA/LTA, and AIC identified a total of 22 microseismic events. Among these events, the time-domain CNN model recognized a total of 11 events, namely the 1st, 2nd, 3rd, 4th, 5th, 11th, 12th, 13th, 14th, 19th, and 20th events. AIC recognized 8 events, namely the 1st, 2nd, 6th, 7th, 8th, 9th, 21st, and 22nd events. STA/LTA recognized 12 events, namely the 6th, 7th, 8th, 9th, 11th, 12th, 13th, 14th, 15th, 16th, 17th, and 18th events. In each microseismic event within this waveform segment, the microseismic waveforms in the 6th and 7th channels are mostly quite distinct and can be easily recognized, which helps meet the event criterion of having microseismic waveforms in at least four channels. For such relatively obvious microseismic events, the number of microseismic events recognized by the three methods is relatively close.

**Fig 9:**
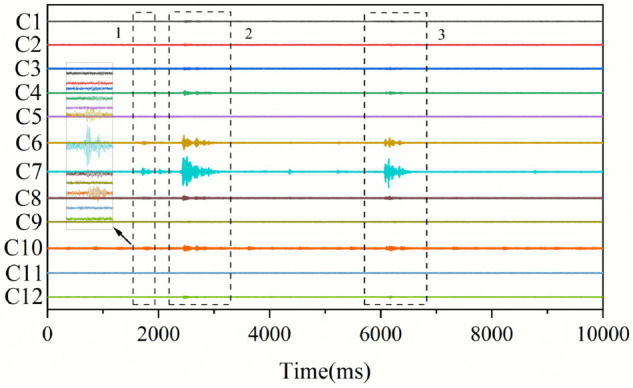
Recognition of weak microseismic events.

In the 15s-long waveform segment in Fig 9, the time-domain CNN model, Fourier CNN model, AIC, and STA/LTA identified a total of 3 events. Obvious microseismic waveforms are relatively easy to recognize. The key to determining whether an event is a microseismic event, meeting the criterion of having microseismic waveforms in at least four channels, lies in the identification of weak microseismic waveforms in other channels. Although there are weak microseismic waveforms in channels 6, 7, and 10 in box a, no microseismic signals were detected in other channels. Therefore, it was not determined that there was a microseismic event in box a. Box 1 represents an event identified by the time-domain CNN model but not by the Fourier CNN model, AIC, STA/LTA, or manual inspection. In this event, no microseismic signals were detected in channels 1, 2, 3, 4, 5, 9, 11, and 12. Weak microseismic waveforms exist in channels 6, 7, 8, and 10, making the identification quite challenging. The time-domain CNN model was able to identify the weak waveforms in channels 6, 8, and 10, thus meeting the event criterion of having microseismic waveforms in at least four channels. However, the Fourier CNN model, AIC, STA/LTA, and manual inspection failed to identify this event. Boxes 2 and 3 represent events jointly identified by AIC, STA/LTA, the time-domain CNN model, the Fourier CNN model, and manual inspection. In these two events, the waveforms in channels 6 and 7 are relatively obvious, making it easy to meet the event criterion of having microseismic waveforms in at least four channels. Therefore, STA/LTA, AIC, and the CNN models can all identify these events. It can be seen that in terms of weak waveform identification, the time-domain CNN model outperforms the Fourier CNN model, AIC, and STA/LTA.

**Fig 10:**
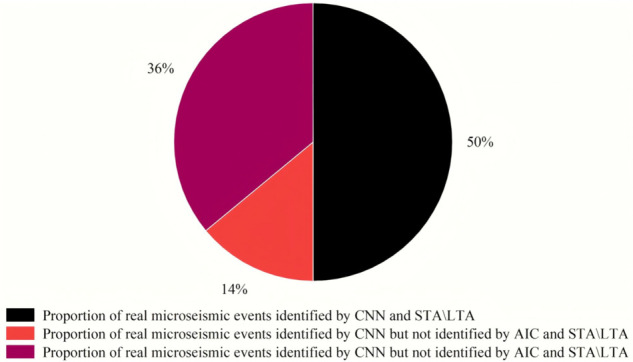
Proportion of the number of identified real microseismic events.

Fig 10 shows the situations of real microseismic events identified by the time-domain CNN model, STA/LTA, and AIC. In one-hour continuous data, the three methods identified a total of 257 real microseismic events (repeatedly identified events are counted as one event). The real microseismic events jointly identified by CNN, STA/LTA, and AIC account for approximately 50%. The real microseismic events identified by STA/LTA or AIC but not by CNN account for about 14%. The real microseismic events identified by the time-domain CNN model but not by STA/LTA or AIC account for approximately 36%. Most of the real events identified by all three methods are relatively obvious real microseismic events, which are relatively easy to recognize. Most of the events identified by the time-domain CNN model but not by STA/LTA or AIC, and those identified by STA/LTA or AIC but not by CNN are real weak microseismic events, which are not easily recognizable by humans. The time-domain CNN model has a better ability to identify weak waveforms than AIC, STA/LTA, and human recognition.

**Fig 11:**
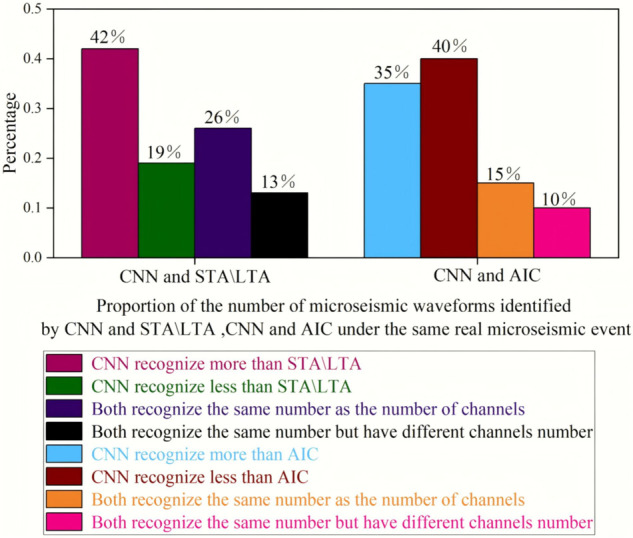
Proportion of the number of microseismic waveforms identified by different methods for the same real microseismic event.

As shown in Fig 11 below, among the 104 real microseismic events jointly identified by the time-domain CNN model and AIC, the proportion of real microseismic events in which the number of microseismic waveforms and the number of channels recognized by both are exactly the same is approximately 10%. The proportion of real microseismic events in which the number of microseismic waveforms recognized is the same, but the number of channels of the microseismic waveforms is not completely the same, is about 15%. The proportion of real events in which the number of microseismic waveforms recognized by the time-domain CNN model is lower than that recognized by AIC is approximately 40%, and the proportion of real events in which it is higher is about 35%. In the waveforms of real microseismic events recognized by AIC, there are many cases where microseismic waveforms are misjudged as background noise. Therefore, the proportion of real events in which the number of microseismic waveforms recognized by the time-domain CNN model is lower than that recognized by AIC is relatively high for the same real microseismic event.

Among the 69 real microseismic events jointly identified by the time-domain CNN model and STA/LTA, the proportion of real microseismic events in which the number of microseismic waveforms and the number of channels recognized by both are exactly the same is approximately 13%. The proportion of real microseismic events in which the number of microseismic waveforms recognized is the same, but the number of channels of the microseismic waveforms is not completely the same, is about 26%. The proportion of real events in which the number of microseismic waveforms recognized by the CNN is lower than that recognized by STA/LTA is approximately 19%, and the proportion of real events in which it is higher is about 42%. In the waveforms of real microseismic events recognized by STA/LTA, there are also many cases where microseismic waveforms are misjudged as background noise. The time-domain CNN model can identify more microseismic waveforms than AIC and STA/LTA, which further proves the superiority of the time-domain CNN model in identifying weak waveforms during hydraulic fracturing of coal seam boreholes.

### Real microseismic events and their waveform recognition situations

To evaluate the applicability of the proposed method for real-time or large-scale microseismic monitoring, we compared the computational efficiency of the time-domain CNN, time-frequency fusion CNN, AIC, and STA/LTA methods.

1. Experimental setup

The hardware and software configurations for efficiency testing were consistent with the model training environment to ensure the reliability of comparative results:

Hardware configuration: CPU (Intel Xeon Gold 6248R, 24 cores), GPU (NVIDIA Tesla V100, 32 GB VRAM), RAM (128 GB DDR4), and storage (1 TB SSD). Software environment: Operating system (Ubuntu 20.04 LTS), deep learning framework (TensorFlow 2.8.0), and data processing libraries (NumPy 1.21.6, SciPy 1.7.3).

Test dataset: One hour of continuous microseismic data (consistent with the dataset in Section Recognition accuracy and recall rate of microseismic events) containing 328 manually verified microseismic events, with a total data volume of 14.4 GB (sampling rate = 4 kHz, 12 monitoring channels).

Evaluation metric: Total computational time, defined as the duration from raw data input to final event recognition results output, including three key stages: data preprocessing (e.g., waveform segmentation, normalization), feature extraction (e.g., time-domain image conversion, time-frequency analysis), and model inference (for CNN-based methods) or statistical calculation (for AIC/STA/LTA). Each method was tested 5 times independently, and the average value and standard deviation of computational time were recorded to reduce random errors.

2. Results and discussion

Table 5 presents the average computational time and standard deviation of each method for processing one hour of continuous data. Traditional methods (AIC and STA/LTA) exhibited the shortest computational time, with average values of 1.8 ± 0.2 s and 2.1 ± 0.3 s, respectively. This is attributed to their simple statistical logic, which only relies on amplitude variation or information criterion calculation without complex feature learning processes. However, their low recall rates for weak signals (Section in Recognition accuracy and recall rate of microseismic events) limit their practical application in high-precision monitoring scenarios.

**Table 5:** Computational time of different methods for one hour of continuous microseismic data.

*Method*	AverageComputationalTime(s)	StandardDeviation(s)
*STA*/*LTA*	1.8	0.2
*AIC*	2.1	0.3
Time−domainCNN	15.3	0.8
Time−frequencyFusion	18.7	1.2

The time-domain CNN model required an average computational time of 15.3 ± 0.8 s, which was slightly longer than traditional methods but still met the real-time monitoring requirements of mine microseismic systems (i.e., computational time < 1% of the data duration, as recommended by industry standards [29]). The time-frequency fusion CNN model had a moderate increase in computational time (18.7 ± 1.2 s) due to the additional time-frequency feature extraction (e.g., short-time Fourier transform, continuous wavelet transform) and multi-channel feature fusion processes.

For large-scale application scenarios (e.g., multi-region integrated monitoring with 50+ sensors), model optimization strategies such as parameter pruning, quantization, and knowledge distillation can be adopted to further reduce computational costs. Preliminary tests show that parameter pruning (retaining 70% of key weights) can reduce the computational time of the time-frequency fusion CNN model by approximately 28% while maintaining a recall rate decrease of less than 3%, which verifies the scalability of the proposed method.

## Conclusion

1. In this study, the CNN model was trained and tested using the time-domain and Fourier images of the hydraulic fracturing data of coal seam boreholes. The time-domain CNN model achieved an accuracy rate of 100% in identifying microseismic events, significantly surpassing the AIC and STA/LTA methods. The integration of computational propagation concepts played a crucial role in enhancing the performance of the model. The features extracted through it enabled the model to capture more comprehensive signal characteristics and improve the identification accuracy.Furthermore, to address the slight class imbalance in the dataset, a weight configuration based on class frequency was introduced into the initial parameters of the model (class_weight[0]=1.014,class_weight[1]=0.996). This further ensured the unbiased learning of the model for background noise and microseismic signals, providing parameter support for high-precision recognition.

2. Since the conversion from the time-domain image to the Fourier image leads to information loss, the time-domain CNN model exhibits higher accuracy and recall rates, indicating that the time-domain image is more suitable as the input data. The computational propagation method enhanced the advantages of this model in data preprocessing. The constructed network and analyzed trends facilitated the distinction between microseismic waveforms and background noise.

3. In response to the deficiencies of traditional algorithms in identifying microseismic signals, the introduction of the time-domain CNN model combined with computational propagation technology effectively differentiated the signals from the noise, achieved precise positioning of fractures, and assisted in subsequent processing. Future work could involve collecting more data, adopting deeper models, and further exploring computational propagation methods to optimize the model.

4. In future research, the geographical coverage of the dataset will be expanded by incorporating microseismic data from different coalfield geological units (e.g., the Carboniferous - Permian coalfields in North China and the Late Permian coalfields in South China). Multi - scenario training datasets will be constructed by integrating rock mechanical parameters (e.g., elastic modulus, Poisson’s ratio) to validate the model’s robustness in heterogeneous media.

## Supporting information

S1 Data Background noise 1. (ZIP)

S2 Data Background noise 2. (ZIP)

S3 Data Background noise 3. (ZIP)

S4 Data Background noise 4. (ZIP)

S5 Data Background noise 5. (ZIP)

S6 Data Background noise 6. (ZIP)

S7 Data Background noise 7. (ZIP)

S8 Data Background noise 8. (ZIP)

S9 Data Background noise 9. (ZIP)

S10 Data Background noise 10. (ZIP)

S11 Data Background noise 11. (ZIP)

S12 Data Effective waveform. (7Z)

S13 Data Picture data. (7Z)
